# Surgery

**Published:** 1860-09

**Authors:** S. W. Gross

**Affiliations:** Lecturer on Surgical Anatomy and Operative Surgery, Philadelphia


### Bi-Monthly Abstracts;

OR, BI-MONTHLY NOVELTIES IN MEDICAL, PATHOLOGICAL, SURGICAL, OBSTETRICAL, PHYSIOLOGICAL, AND CHEMICAL SCIENCE.

### SURGERY.

BY S. W. GROSS, M.D.,

LECTURER ON SURGICAL ANATOMY AND OPERATIVE SURGERY, PHILADELPHIA.

I.—*A Statistical Examination of the Operation of Deligation of the Primitive Iliac Artery, embracing the Histories, in abstract, of Thirty-two Cases.* By STEPHEN SMITH, M.D., Surgeon to Bellevue Hospital, New York. (Amer. Journal of Med. Sciences, July, 1860.)

DR. SMITH having had occasion to ligate the primitive iliac artery for aneurism of the external iliac, makes his case the basis of a very valuable contribution to the literature of deligation of arteries, and gives, in abstract, the histories of thirty-two recorded cases. This difficult operation was first performed by Dr. Gibson in 1812, and the second instance was under the charge of Dr. Mott in 1827, the patient being perfectly cured on the forty-fifth day. In this connection it is interesting to remark that fifteen of the thirty-two cases have been in the hands of American surgeons, and that in three the operation resulted in recovery.

The indications which have called for the ligation of the primitive iliac artery are represented by Dr. Smith under the following heads:—1. For the arrest of hemorrhage. 2. For the cure of aneurism. 3. For the cure of pulsating tumors, which proved to be malignant growths. 4. For the prevention of hemorrhage in the removal of a morbid growth.

1. For the arrest of hemorrhage, we find eleven cases recorded, of which ten proved fatal, the mortality being 91 per cent. The earliest period of death after the operation was four hours; the latest, the twenty-fifth day—the average being eight days. The cause of death in four instances was secondary hemorrhage; in five, immediate exhaustion; in one, peritonitis. In two of these cases the procedure was instituted for wounds involving, in one, the external iliac, in the other, the internal epigastric; in one for secondary hemorrhage from the stump after amputation of the thigh; in two from rupture of an aneurism; in three from hemorrhage after deligation of the external iliac; in two from incisions into aneurismal tumors; and in one from rupture of the internal iliac in an attempt at its ligation for aneurism of the gluteal artery. In the case of recovery the operation was performed for hemorrhage after ligature of the external iliac.

2. In the second group fifteen cases are reported, five resulting in recovery, the mortality being 66⅔ per cent. The patient of Dr. Mott was permanently cured and is still alive, the artery having been ligated for aneurism of the external iliac. The case of Dr. Smith comes under this division, the right femoral and external iliac arteries being the seat of the aneurism. The patient died from hemorrhage on the forty-eighth day. Of these fifteen cases, all but one were males; the right external iliac was affected in eight instances, the left in six, and in one case the side is not given. The cause of death in the ten fatal cases was, in two, hemorrhage; in two, exhaustion; in one, gangrene of the leg; in one, gangrene of the aneurismal sac and the surrounding parts; in one, erysipelas; in one, dysentery; in one, suppuration of the sac; and in one, peritonitis.

3. The common iliac was ligated four times for malignant tumors simulating aneurisms. In the case of Mr. Guthrie, his colleagues agreed with him that it was one of aneurism, but the tumor afterwards proved to be malignant. This was the only one of the four patients who recovered.

4. For the prevention of hemorrhage two cases are recorded, one being for encephaloid disease, the other for aneurism by anastomosis, in a child six weeks old. Both terminated fatally, the causes of death being, respectively, exhaustion and abscess of the knee.

In reviewing the facts contained in this paper, it appears that in seventeen cases the right primitive iliac was ligated, and in thirteen, the left primitive iliac, the operation being required, directly or indirectly, twenty-four times for aneurism. The tumor involved the right external iliac eleven times, the left seven; thus showing the greater liability of the former to aneurism. That males are more exposed to the exciting causes of aneurism is verified by the fact that twenty-seven of these cases occurred in males, and three in females; the sex not being given in the remaining two; the ages of the patients varying from six weeks to fifty-nine years. The date of separation of the ligature is mentioned in twelve cases, the shortest period being eight days, the longest thirty-six days; the average being twenty-three days. In one case the silver wire was used; and in another the ligature was composed of catgut; the latter came away on the eighth day and was found to be dissolved. That the operation of casting a ligature around the common iliac is one of the most unsuccessful in all surgery, is shown by the fact of twenty-five of the thirty-two cases having died, being a mortality of about $78\frac{1}{8}$ per cent.

From numerous trials upon the dead subject, Dr. Smith advises the following incision as affording the most ready access to the artery:—

Commence the incision just anterior to the extremity of the second false rib (eleventh) and terminate it just above the internal ring by a sharp curve inward of one inch; this incision will be about seven inches in length, and will pass about an inch and a half within the anterior superior spinous process; the curve at the lower extremity will allow the most perfect freedom in the elevation of the peritoneum, and the complete exposure of the artery.

II.—*A Case of Stone in the Bladder; High Operation; unsuccessful Result.* By JAMES R. WOOD, M.D., Surgeon to Bellevue Hospital. (American Med. Times, vol. i., June 2, 1860.)

Dr. Wood performed the supra-pubic operation of lithotomy upon a man, fifty-eight years of age, the method being preferred on account of the remains of an old stricture, and a greatly enlarged prostate. A calculus, about the size of a pigeon's egg, was removed, and during the operation the peritoneum was ruptured, allowing the protrusion of a portion of the small intestine. The wounded membrane and transverse fascia were united by two silver sutures, and the wound in the bladder by two more, and the external incision was closed in the same way, and a flexible catheter secured in the bladder. On the second day after the operation, the patient died. There were slight signs of peritonitis; the peritoneum was found to have been ruptured at the point of its reflection from the bladder, as well as where it is reflected on to the abdominal wall, and that portion which had been tacked together with the sutures was united. There was no extravasation of urine, but the urinary apparatus was very extensively diseased.

Our object in adverting to this case is to present our readers with a few points in regard to the high operation as performed in New York. Dr. Wood has collected ten cases in which this method has been practiced in that city, and appends to his paper brief histories of each. Professor Parker has operated four times, the patients being adults, and three females. One case was fatal four days after the operation, and occurred in a male subject. Dr. Krackowitzer has had three cases, two of which were in young children, and all died in from ten hours to three days after operation. Dr. Noeggerath operated successfully upon a boy, eight years of age, and removed a large calculus. Dr. Hewit, formerly of the United States army, operated upon a young man, who made a rapid recovery. Of these ten cases we, therefore, find that only five were successful, adding more evidence against an operation which is known to every one to be fraught with great danger.

III.—*New Operation for Subunguial Exostosis.* By DR. DEBROU, of New Orleans. (Gazette Hebdomadaire, June 1, 1860.)

Exostosis of the last phalanx of the great toe, although a rare affection, finally gives rise to much pain and impediment in walking. It is of a fibro-osseous nature, and may be situated on the distal phalanx of any of the toes or fingers, although the great toe is most obnoxious to this excrescence. The cases of M. Debrou, five in number, show that the pedicle is always situated on the summit of the phalanx, while its base lifts up the nail, and contains fibrous tissue, covered by a red membrane of a mucous appearance, which bleeds readily.

Cauterization, as practiced by André, and excision, as performed by Dupuytren, Liston, and other surgeons, by means of a stout bistoury, aided, if necessary, by the chisel, and disarticulation of the phalanx, are all, in M. Debrou's opinion, unworthy of reliance. The danger in excising the excrescence on a level with the bone, is its liability to return, as has been observed in several instances; and to overcome this difficulty the author has devised the following operation. The nail is split from before backward with a pair of sharp scissors, and its two halves are torn out. An incision is then made upon the back of the phalanx, which is prolonged upon the sides of the tumor, so as to circumscribe it and lay bare the summit of the phalanx. By means of cutting pliers the bone is divided in front of its base, thus getting rid of the entire exostosis, with a sound portion of the bone, which effectually prevents its return. The base of the phalanx being left in place, the articulation and tendons are preserved. When the exostosis affects the last phalanx of the small toes and the fingers, we should not hesitate to disarticulate, as the phalanges are of so small dimensions that it would be useless to preserve a part of them.

IV.—*Application of the Button Suture to the Treatment of Varicose Dilatation of the Veins.* By NATHAN BOZEMAN, M.D., of New Orleans. (New Orleans Medical and Surgical Journal, July, 1860.)

For the radical cure of varix of the saphenous vein, Dr. Bozeman recommends the application of the button suture, in the same way as in the treatment of vesico-vaginal fistule. He thinks the advantages of the silver wire over the silk thread are, that it is more innocuous, and that it divides the vein more readily and effects its occlusion with less risk. The button, acting as a shield, affords the skin over the vein protection against undue pressure. The author, in operating, prefers the patient being placed in the erect position, for the reason that the vein is distended, and the needle can, therefore, be carried around it with more facility, and with less danger of penetrating its coats. He reports two cases, in which the button suture was tried, one being completely cured, the other greatly relieved. He also operated successively in the same way upon a large varicocele, the suture apparatus being removed on the seventh day, when the veins were thoroughly consolidated.

V.—*On Myeloid Tumors.* By R. P. HOWARD, M.D., Professor of Clinical Medicine in McGill College. (Pamphlet, 1860, and Ranking's Half-Yearly Abstract, No. xxxi., 1860.)

From the journal above quoted we extract entire the conclusions of Dr. Howard respecting myeloid tumors. Our own opinion is that these growths are malignant, and partake most probably of the nature of encephaloid:—

1. Myeloid tumors may occur with about equal frequency in both sexes.

2. Local injury was the apparent exciting cause of the growths in about one-fourth the entire number, and in thirteen of the thirty-eight cases no cause could be assigned.

3. Myeloid tumors occur chiefly before thirty years of age, for 76 per cent. of the cases were under that age, and 90 per cent. were under forty; they may occur at as advanced an age as seventy-four.

4. While myeloid and cancerous tumors are of about equal frequency under twenty, myeloid are more frequent than cancerous in the ratio of forty-seven to twenty at the decade between twenty and thirty.

5. The bones are of all parts of the body most prone to myeloid growths; in about three-fourths of the cases it is the long bones which are implicated; and in perhaps all cases the disease begins in and is confined to the articular extremities of such bones.

6. The condyle of the femur is the part of the body most obnoxious to these tumors, probably the head of the tibia next, and the superior maxilla next.

Several other localities exhibit about equal susceptibility, viz.: the head of the humerus, the head of the fibula, and the inferior maxilla.

7. No bone is probably exempt.

8. Of the soft parts, it is chiefly the fibrous tissues, and especially those in proximity to bones and articulations, that are most liable to myeloid growths; but they have been rarely seen in the lungs, in the neck, in a lymphatic gland, and in the mamma; in the last site, it was probably associated with cancer.

9. These growths very seldom extend into an articulation; this event having been noticed only twice in twenty-five cases, in which the disease occupied the articular extremity of long bones: even should the articulation be entered by the growth, the cartilages are not usually implicated.

10. Secondary inflammation occasionally is excited in the contiguous articulation, but it is of an adhesive rather than a suppurative character.

11. Data are wanting to determine the average duration of life when myeloid tumors are not interfered with.

12. The average duration of life after removal of myeloid tumors far exceeds its average duration after removal of cancerous; a large proportion of the subjects of the growth were alive five years and eight months subsequently to the operation.

13. Of two deaths which followed removal of the tumor at the respective intervals of five and two years, the cause was accidental and not connected with the disease.

14. So far as we know, pure myeloid disease exhibits little proneness to recur after removal, there being only one instance yet recorded of that event;* but, then, in only half the cases collected, is the subject of recurrence mentioned, and in many others sufficient time had scarcely elapsed to justify any opinion.

15. While medullary cancer recurs, on the average, in seven months, and scirrhous cancer in fourteen, myeloid tumor, in eighteen instances, had not returned after an average interval of twenty-six months, and in twelve of these, or two-thirds, the period of non-recurrence was three years and five months.

16. Myeloid may exceptionally recur as myeloid both locally and in remote organs; the lymphatics enjoying immunity, and there being no cachexia.

17. It may coexist in an external part, in the lungs and in a lymphatic gland, and even prove fatal without the presence of constitutional cachexia.

18. The same growth may comprise both myeloid cells and so-called "cancer cells," although in general appearance resembling myeloid tumors, and be succeeded by similar compound tumors in the lungs and spine, with marked cachexia.

19. A tumor apparently myeloid, even on microscopic examination, may be followed after removal by genuine open cancer in the vicinity of the original tumor.

20. A tumor composed chiefly of fibro-plastic structure, and partly of myeloid, may be attended with enlargement of the glands, and, when removed, be rapidly

---

* Dr. Howard has purposely omitted some cases of myeloid disease of the maxilla, which reappeared after removal, apparently in consequence of having been only partly excised.

succeeded by cancer at the site of removal and in the lungs, the glands, though enlarged, not being cancerous.

21. Of forty-two examples of growths apparently myeloid, two of which, however, contained cancer-cells, and one fibro-plastic elements, there were five in which the growth either recurred after the removal, or had involved remote internal organs.

VI.—*Fistulous Ulcer in front of the Larynx.* By JOHN WATSON, M.D., Surgeon to the New York Hospital. (American Medical Times, vol. i. No. 1, 1860.)

Dr. Watson reports two cases of fistule, connected with a synovial bursa, near the hyoid bone, which existed in front of the larynx, both occurring in females. The history of these cases is almost identical, and a short abstract of one will suffice to point out the nature and treatment of this little-known affection. A young lady, aged twenty, for two or three years had been troubled with a papillary ulcer over the thyroid cartilage, which was the seat of a constant discharge of a glutinous fluid resembling inspissated synovia. A delicate probe introduced into the orifice was readily passed up in the median line of the neck for about an inch under the integuments, in front of the thyro-hyoid ligament, and rested at the lower border of the hyoid bone. On removing the probe, and manipulating the parts, a delicate indurated track could readily be detected. Several unsuccessful operations, with a view to close the orifice, had been performed, when Dr. Watson advised injecting the fistule first with water in order to cleanse it—afterwards throwing in a solution containing five grains each of corrosive sublimate and hydrochlorate of ammonia to the ounce of water. A single application arrested the discharge, and in a few days the fistule was cured.

VII.—*On the Treatment of Purulent Ophthalmia of Infants and Children.* By PROFESSOR STELLWAG VON CARION. (Oesterreich Jahrb. für Kinderheilkunde, und Zeitschrift für Medicin, Chir. und Geburtshülfe, xiv. 2, 1860.)

Professor Stellwag recommends the application of pressure to the eyes by means of a suitable bandage in cases of purulent ophthalmia of infants, and states that he has obtained far more favorable results from this mode of treatment than from any of the methods usually employed. It is particularly indicated in those cases where the inflammation has reached a high degree, and where, in consequence of it, the swelling is considerable. If properly applied, it not only protects the eye from glaring light, from the introduction of foreign matters into the conjunctival sac, and hinders the patient from rubbing or touching the swollen lids, but it reduces also the serous swelling of the parts, and keeps down the growth of granulations in a very marked degree. It prevents the accumulation of purulent matter, as it presses the two layers of the conjunctival sac firmly against each other, and forces the secretions to come out between the edges of the eyelids. The dressing consists of a compress of fine charpie, which is equally distributed over the closed eyelids, and kept in position by an elastic bandage making more or less pressure on the parts. As the proper material for such a bandage, Professor Stellwag advises a strip of the finest flannel, two inches and a half wide and eight inches long, which is cut obliquely across the fibres of the texture, and to the two tapering ends of which tapes of about half an inch in width are attached; these tapes are carried around the head, and then tied into a knot on the forehead. A very good substitute for charpie is carded cotton, which, on account of its greater elasticity, may be in many cases even preferable to the former. The dressing must be frequently changed, in order to remove the pus which has accumulated underneath it, and to cleanse the eye, as the secretion on drying converts the compress into a hard mass with an uneven surface, unfit for maintaining an equal pressure. It is well to soak the compress in some astringent solution before applying it. As cotton absorbs moisture with more difficulty than charpie, a layer of moistened charpie may be spread over the eyelids, and the cotton applied over it. When the disease is of a milder character and the amount of the secretion but small, the author recommends lead-water as best adapted to the purpose; in cases, however, where the secretion is abundant, and particularly when it is of an unhealthy character, as in diphtheritic conjunctivitis, he decidedly prefers a solution of nitrate of silver—five to ten grains to one ounce of water. If there is great relaxation of the parts, with a large amount of mucous secretion, he makes use of a solution of one drachm of the sesquichloride of iron to the ounce of water. Great care must be taken that the eyelids are in their normal position underneath the compress, and the dressing must be applied with greater caution, if the cornea is in an ulcerated or sloughing condition. If the swelling of the eyelids and of the conjunctiva has almost entirely disappeared, the inflammation has subsided, and only a relaxed condition of the parts with an insignificant amount of mucous secretion remains, there is no further necessity for applying the dressing, and the cure may be completed by the application of slighly stimulating and astringent remedies in the form of fomentations and collyria. Granular conjunctivitis is never observed after this treatment. The bandage should not be left off at once, but removed gradually, and in a darkened room, so as to accustom the patient by degrees to a stronger light.

Professor Stellwag believes that the application of pressure, in combination with astringent remedies, might be used also with advantage in granular conjunctivitis of long standing.

VIII.—*On Amputation at the Hip-Joint.* By DR. JULES ROUX. (Gazette Hebdomadaire, May 4th and 11th, 1860.)

In our last issue we presented our readers with a short abstract of a paper by Dr. Roux, on secondary amputations after gunshot injuries of the bones; and in a recent communication to the Academy of Sciences he reported six cases of coxo-femoral disarticulation, performed in his wards at the St. Mandrier Hospital, at Toulon, upon the sailors and wounded soldiers of the Italian army. Two of these cases resulted in death, but both sailors had comminuted fractures of the upper third of the thigh-bone, with laceration of the femoral vein and extensive injury of the soft parts. The patients succumbed, one in five, the other in eight hours; thus adding confirmatory evidence against the success of primary amputation at the hip. In the four successful cases, the operation was secondary or consecutive, and the cure was complete on the seventy-first, sixty-sixth, fifty-eighth, and seventy-seventh days after the operation. All the patients, except the two fatal ones, were placed fully under the influence of chloroform, and the bleeding vessels, including the femoral and larger veins, required at least sixteen ligatures. The dressings consisted in the application of ten twisted sutures, with adhesive strips, and a piece of greased lint over the wound. A drainage tube or tent was retained in the parts for twenty-five or thirty days. Light but nourishing diet, Bordeaux wine, a decoction of quinine and aconite, with attention to the bowels and secretions, cleanliness and ventilation, constituted the after-treatment.

Dr. Roux also gives a statistical table of the cases of coxo-femoral disarticulation occurring in the French marine hospitals. It contains twelve cases, including his own, of which seven terminated fatally. Five of these operations were primary, or performed several hours or days after the accident, and all were unsuccessful; four were secondary or mediate, forty-two days to seven months intervening between the accident or disease and date of operation, and three were cured; three were ulterior or consecutive, the operation being performed one or several years after the receipt of the disease or injury, and only one resulted in death.

The author lays it down as a rule, that comminuted fracture of the thigh-bone, in any portion of its extent, rarely requires amputation; and insists, when the fracture is seated in the upper third of the bone, that amputation at the hip-joint should not be performed. In support of his opinion he gives a brief account of twenty-one cases received into the St. Mandrier Hospital after the Italian war, in which the superior third of the thigh-bone had been fractured by balls, and in all the fracture had become consolidated. Secondary coxo-femoral disarticulation was practiced successfully in one case, six months after the occurrence of the injury, and the remaining twenty were cured. Amputation should, therefore, as a rule, be confined to those cases where the two lower thirds of the bone are injured; and if after several months a cure is not effected, and amputation becomes necessary, whatever may be the portion of bone fractured, coxo-femoral amputation should be preferred, as in this way all the diseased bone is gotten rid of. The longer the operation is delayed, the greater is the chance of recovery.

IX.—*On the Treatment of Stricture of the Urethra.* By PROFESSOR SIGMUND, of Vienna. (Prager Vierteljahrschrift für die Praktische Heilkunde, xvii. Band 2, 1860.)

In cases of urethral stricture, Dr. Sigmund prefers the treatment by dilatation, and in his clinic has obtained most excellent results from the method "*aux bougies multiples*," recently recommended by Amussat. The treatment is commenced by introducing a very slender, thread-like bougie, which is retained during the first twenty-four hours. On the following day, a second instrument is passed along the side of the first one; on the third day, another, and so on, until five or six bougies have been introduced. Having arrived at this point an ordinary larger bougie is made use of. This method, in Dr. Sigmund's opinion, possesses the advantage of giving the patient but slight pain, and of rendering the discharge of urine much easier, on account of the great flexibility of the bundle.

X.—*On Fracture of the Surgical Neck of the Scapula.* By JURIAH HARRISS, M.D., Professor of Physiology in the Savannah Medical College. (Savannah Journal of Medicine, July, 1860.)

Five cases of this rare accident have come under the observation of Dr. Harriss, and two within his own practice. Surgical writers are not agreed as to the possibility of the occurrence of such an injury, but the discrepancy among them evidently arises from a misunderstanding as to what portion of the bone is injured. The surgical neck, or that part of the scapula behind the coracoid process, and not the anatomical neck, is always the seat of the lesion, the fracture passing between the anterior root of the acromion process and the rim of the glenoid cavity.

The case reported by Dr. Harriss occurred in a negro boy, twelve years of age, who fell from a fence upon his left shoulder. The symptoms consisted in great prominence of the acromion, flattening and deformity of the shoulder, the distance from the inner margin of the axilla to the acromion process was increased, and the coracoid process and head of the humerus were more prominent than on the sound side. The patient could elevate the limb and move it from side to side, and was able to carry his hand to the opposite shoulder, the elbow being in contact with the side of the chest. Crepitation could readily be elicited, and by forcing the shoulder upward, outward, and backward, its rotundity was restored, but again became flattened on ceasing the force. The head of the humerus could not be felt in the axilla, a symptom which was also absent in two other cases seen by the author. There was no great pain nor swelling.

The treatment consisted in fixing the scapula, and keeping the arm pressed upward, backward, and outward, by appropriate apparatus. At the end of the seventh week the fracture was united.

XI.—*On a little known Variety of Syphilitic Aphonia.* By M. P. DIDAY. (Gazette Médicale de Lyon, and Gazette Hebdomadaire, No. 19, 1860.)

In this paper M. Diday describes what he terms as *secondary aphonia*, to distinguish it from the hoarseness present in laryngeal phthisis in subjects in the last stages of an inveterate syphilis. His conclusions are based upon twenty cases, of which the following is a summary:—

Between the third and sixth months from the appearance of the primary affection, the patient, without having been exposed to any exciting causes, or having symptoms of coryza, angina, or bronchitis, perceives that the voice has lost its ordinary sonorousness; an alteration which increases so rapidly that in a few days he cannot speak above a whisper. Apart from the change of volume, the other functions of the vocal organs remain perfect. Pronunciation is clear and distinct, the respiration is unaltered, and there is neither pain, cough, dyspnœa, nor fever. When this condition becomes once established, it will, in all probability, continue indefinitely, unless a proper course of treatment is instituted, for there is little or no tendency to a spontaneous cure. This disease is more common in syphilitic subjects than is generally supposed, and patients ascribe it to the ordinary causes of hoarseness. On account of the period of its appearance, the average being four months, this aphonia should be classed among the secondary affections; and it is sometimes accompanied by mucous tubercles of the tonsils.

In view of the absence of pain, malaise, and the great promptness of the cure, M. Diday is disposed to consider this secondary aphonia a nervous affection, due to paralysis of the muscles which endow the borders of the orifice of the glottis with their power of vibration. Under the influence of eight to ten centigrammes of the protiodide of mercury a day, the affection is changed in two days, and six or eight days later a cure is effected.

XII.—*On the Treatment of Staphyloma by Ligature, according to Borelli's Procedure.* By M. ANCELLON. (Bull. de Thérapeutique, and British and Foreign Med.-Chir. Review, July, 1860.)

Our attention having been drawn to the ligature treatment of staphyloma, by the report of M. Ancellon, quoted in the British and Foreign Review, we were induced to refer to the volume of the Transactions of the Ophthalmological Congress, held at Brussels in 1857, and found a paper on the treatment of staphyloma, by Dr. Jean-Baptiste Borelli, of Turin. Although several years have elapsed since the publication of this memoir, we still think that a short abstract of this new treatment will prove acceptable to our readers, especially as attention has again been directed to the subject by M. Ancellon.

Dr. Borelli is convinced that the treatment of staphyloma by the ligature possesses the advantages of preserving the globe of the eye with little deformity, it permits the more easy adaptation of an artificial eye, and in certain cases the condition of the organ is such that a fair chance for sight is offered by the formation of an artificial pupil; moreover, a relapse, as after other procedures, is impossible. This operation is merely a modification of that of Celsus, and for its performance are required two long and fine pins—such as insect pins—a silk ligature, and a pair of forceps. An assistant elevates the upper lid, while the operator controls the lower, at the same time fixing the globe of the eye, as in the operation for cataract. He then traverses the base of the staphyloma with the pins in the form of an X, and carrying the ligature behind the pins strangulates the mass. The ends of the thread are confined to the cheek by means of a strip of adhesive plaster. The lids are protected by interposing between them and the pins pieces of greased lint; the orbital region is covered with a pledget of charpie covered with cerate, and over all a compress is placed, confined in its position by a roller or handkerchief. That this operation is not painful, is shown from the fifteen cases of Dr. Borelli, and from those occurring in the practice of MM. Ancellon and Guépin.

On removing the dressings on the third day, the pins, with the thread and strangulated staphyloma, are generally found detached from the eye. The dressings of greased lint are renewed every twenty-four hours, and on the eighth day the cicatrix will be found to be quite firm. By the twentieth day after the operation the patient can resume his usual occupation.

